# Unexpected insights for anti-EGFR cancer therapy

**DOI:** 10.18632/oncotarget.5115

**Published:** 2015-08-07

**Authors:** Philippe Depeille, Robert S. Warren, Jeroen P. Roose

**Affiliations:** Department of Anatomy, University of California, San Francisco, San Francisco, California, USA

**Keywords:** Chromosome Section, colorectal cancer, therapy, Ras, EGFR

Colorectal cancer (CRC) is a major cause of morbidity and mortality. It accounts for over 9% of all malignancies, making it the third most common cancer worldwide and second most common cause of death in the developed world. Treatment of metastatic colorectal cancer (mCRC) is largely palliative, making potentially curative therapy via molecular inhibition of specific cellular targets in CRC a highly sought-after goal. However, the complexity and non-intuitive nature of cancer signaling pathways have made the development of effective targeted drugs a major challenge.

One target that has been the focus of considerable basic and clinical investigation in CRC is the Epidermal Growth Factor Receptor (EGFR). Despite widespread EGFR expression in CRC, clinical trials have shown modest benefit from the use of anti-EGFR blocking antibodies (panitumumab or cetuximab) [1]. In the trial that led to FDA approval of cetuximab in mCRC, response rate to single agent cetuximab in patients who had progressed on prior chemotherapy was only 11% [2]. Subsequently it was shown that tumors with activating mutations in KRAS (*KRAS^MUT^*) do not benefit from EGFR blockade [3]. Given the ubiquitous expression of EGFR in CRC, why does such a large subset of patients fail to benefit from anti-EGFR antibodies? The most straightforward explanation for failure of anti-EGFR therapy in *KRAS^MUT^* CRC is that the constitutive activity of KRAS^MUT^ bypasses activation of the EGF receptor lying upstream. But this does not explain the primary resistance in patients whose tumors have wild-type KRAS. The finding of heterogeneous effects of anti-EGFR treatment in wild-type tumors as well as in those with different subsets of KRAS mutations [4] emphasizes the need to improve our understanding of the mechanisms, which underlie both responsiveness and primary resistance to these therapies.

The oncogenic mutation in *KRAS* traps this small GTPase in the active GTP-bound state, leading to strong pro-proliferation and anti-differentiation signals. Illustrative of the potency of RAS, 30% of all metastatic cancers carry somatic *KRAS* mutations, such as *KRAS^G12D^*, and approximately 40% in CRC [1,4]. Of note, the quest for small molecules that revert the trapped active KRAS back to baseline activity has been ongoing for decades and has recently gained momentum, but has not yet been successfully completed.

Recent pancreatic cancer studies challenge the most common explanation for insensitivity to EGFR blockade and indicated that EGFR signals can modulate tumor growth in *KRAS^Mut^*-driven pancreatic ductal carcinoma (PDAC) in mice [5]. In the clinic Erlotinib is beneficial for a limited number of PDAC patients [6]. Thus, *KRAS^MUT^* does necessarily operate entirely autonomously but can be influenced by EGFR signal input. Mechanistic insights into this phenomenon were lacking, which we set out to investigate in the context of CRC and the Ras guanine nucleotide exchange factors (RasGEFs) that activate RAS by loading GTP onto this small GTPase.

**Figure 1:**
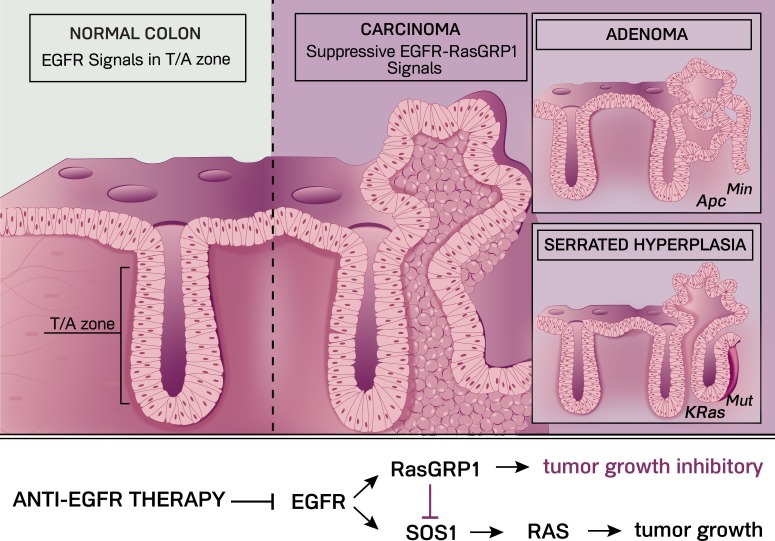
In the normal colon (far left), EGFR signals in the transit-amplifying (T/A) zone drive regulated expansion of the colonic epithelial progenitor cells that subsequently differentiate. We found that EGFR-RasGRP1 signals play a growth suppressive role in *KRas^G12D^*-driven serrated hyperplasia and in adenomas caused by the *Apc^Min^* mutation, both of which can progress to full carcinoma. Illustration by Anna Hupalowska.

In our recent publication [7], we reported that two structurally distinct types of RasGEFs, SOS1 and RasGRP1, are co-expressed in both normal intestinal epithelial cells and in CRC cells. In line with the indications in PDAC, we observed that EGFR signals in CRC cells with *KRAS^MUT^* result in even more RAS activation, arguing that RasGEFs load more RAS molecules with GTP following EGF stimulation. Indeed, we found that both SOS1 and RasGRP1 are activated downstream of the EGFR (Figure 1). Quite surprisingly, SOS1 and RasGRP1 have opposing functions here. Biochemically, RasGRP1 limits the amount of EGFR-SOS1-RAS signals. Biologically, knockdown of RasGRP1 expression in *KRAS^MUT^* CRC cells leads to increased tumor growth in a xenograft mouse model. In contrast, knockdown of SOS1 expression results in the opposing phenotype with decreased KRAS activation and reduced CRC growth.

Multiple pathways in normal colonic epithelium can transform into a full carcinoma, such as through serrated hyperplasia- or adenoma-intermediaries (Figure 1). Mouse models with intestinal expression of *KRas^G12D^* or of *Apc^Min^* (a mutated form of the tumor suppressor *Adenomatous Polyposis Coli* that results in oncogenic Wnt signaling) have been widely used to better understand intestinal carcinogenesis. *KRas^G12D^* leads to serrated hyperplasia of the colonic epithelium and *Apc^Min^* in mice drives adenomas, mostly in the small intestine and sometimes in the colon (see Figure 1). In both models we observed that deletion of *Rasgrp1* alleles results in an exacerbation of the *KRas^G12D^*- or *Apc^Min^*-driven pathology, providing genetic proof for the existence of a tumor suppressive role for RasGRP1 (Figure 1). In the case of *KRas^G12D^*, removal of Rasgrp1 resulted in a marked increase of the serrated hyperplasia, whereas *Apc^Min^* mice with deleted Rasgrp1 revealed increases in colonic adenomas and decreased overall survival [7].

How do our studies relate to human CRC? Mining databases and analyzing CRC patient samples, it became clear that not all CRC express the same level of RasGRP1 and that expression is lower in human colon adenomas compared to healthy colonic tissue. Significantly, CRC patients with relatively high levels of RasGRP1 in tumors demonstrated better clinical outcome compared to those with low levels of RasGRP1 in tumors [7]. In sum, our study reveals a novel growth inhibitory function for RasGRP1 downstream of the EGFR in CRC (Figure 1). We anticipate that these findings will prove of relevance to anti-EGFR therapy in the future as we hypothesize that anti-EGFR therapy may lead to unwanted inhibition of this inhibitory function when CRC tumors express significant levels of RasGRP1 protein.
